# Minor gait impairment despite white matter damage in pure small vessel disease

**DOI:** 10.1002/acn3.50891

**Published:** 2019-09-16

**Authors:** Sofia Finsterwalder, Max Wuehr, Benno Gesierich, Anna Dietze, Marek J. Konieczny, Reinhold Schmidt, Roman Schniepp, Marco Duering

**Affiliations:** ^1^ Institute for Stroke and Dementia Research University Hospital LMU Munich Munich Germany; ^2^ German Center for Vertigo and Balance Disorders DSGZ Department of Neurology University Hospital LMU Munich Munich Germany; ^3^ Department of Neurology Medical University of Graz Graz Austria; ^4^ Munich Cluster for Systems Neurology (SyNergy) Munich Germany

## Abstract

**Objective:**

Gait impairment is common in patients with cerebral small vessel disease (SVD). However, gait studies in elderly SVD patients might be confounded by age‐related comorbidities, such as polyneuropathy or sarcopenia. We therefore studied young patients with the genetically defined SVD CADASIL. Our aim was to examine the effects of pure SVD on single and dual task gait, and to investigate associations of gait performance with cognitive deficits and white matter alterations.

**Methods:**

We investigated single task walking and calculatory, semantic, or motoric dual task costs in 39 CADASIL patients (mean age 50 ± 8) using a computerized walkway. We obtained 3.0T MRI and neuropsychological data on processing speed, the main cognitive deficit in CADASIL. Spatiotemporal gait parameters were standardized based on data from 192 healthy controls. Associations between white matter integrity, assessed by diffusion tensor imaging, and gait were analyzed using both a global marker and voxel‐wise analysis.

**Results:**

Compared to controls, CADASIL patients showed only mild single task gait impairment, and only in the rhythm domain. The semantic dual task additionally uncovered mild deficits in the pace domain. Processing speed was not associated with gait. White matter alterations were related to single task stride length but not to dual task performance.

**Interpretation:**

Despite severe disease burden, gait performance in patients with pure small vessel disease was relatively preserved in single and dual tasks. Results suggest that age‐related pathologies other than small vessel disease might play a role for gait impairment in elderly SVD patients.

## Introduction

Gait impairment and cognitive deficits are common symptoms in cerebral small vessel disease (SVD) and a major cause of loss of independence.^1^, ^2^ For mobility capabilities, both symptoms bear a high risk for falls and fractures, specifically in the elderly.^3^ Senile, vascular gait impairment in sporadic SVD patients has been characterized by a reduction in gait velocity,^4^, ^5^, ^6^, ^7^, ^8^ reduction in stride length,^4^, ^6^, ^7^ and increased double support times.^4^, ^6^ However, the etiology of gait impairment in SVD is still debated. The traditional view holds that lesions in strategic white matter tracts have a detrimental effect on supraspinal locomotor control.^4^, ^6^, ^9^, ^10^ This view is challenged by studies showing that gait control can also be affected by age‐related instability due to degenerative musculoskeletal impairments, for example, joint problems, sarcopenia, or polyneuropathy.^3^ A complementary view is that cognitive deficits are a major cause of gait disturbances and falls.^11^ The notion is based on experiments using cognitive dual‐tasking, in which participants perform an attention‐demanding task while walking.^12^ Gait performance deteriorates under this condition in healthy subjects^13^ and especially in cognitively impaired subjects.^14^, ^15^, ^16^, ^17^, ^18^ The underlying hypothesis is that walking, that is, planning and execution of movements, postural control, motor coordination, and the secondary cognitive task compete for the same limited cognitive resources. While gait difficulties can be cognitively compensated during single task walking, this compensation mechanism is disrupted or limited by a secondary cognitive task. Thus, dual task walking can pronounce or even uncover gait deficits that are not obvious while walking only.^3^, ^18^

Results of previous studies on gait impairment in SVD are based on sporadic SVD patients or individuals with mild cognitive impairment (MCI) aged 60 years or older.^4^, ^6^, ^7^, ^9^, ^10^, ^18^, ^19^ A potentially crucial limitation of these studies is the confounding by age‐related co‐pathologies, such as affected biomechanics, sarcopenia, or disturbed sensory feedback (vision, proprioception). One approach to overcome these limitations is to explore the effect of dual‐tasking on gait in a model disease of pure SVD without confounding pathology. We therefore studied patients with cerebral autosomal dominant arteriopathy with subcortical infarcts and leukoencephalopathy (CADASIL), a genetically defined, pure form of SVD. CADASIL is characterized by an early disease onset between 35 and 50 years.^1^ Conditions typically impacting on gait performance in elderly subjects, such as musculoskeletal constraints, joint abrasion, polyneuropathy, Alzheimer‐type changes, or other neurodegenerative pathology,^20^ and normal pressure hydrocephalus^21^ are uncommon in CADASIL patients.

SVD‐related white matter alterations can be assessed using diffusion tensor imaging (DTI). DTI scalar measures are sensitive markers for SVD progression^22^ and show a stronger association with gait decline than conventional SVD markers, such as white matter hyperintensities (WMH), lacunes, and microbleeds.^9^

The aim of the present study was to investigate the effect of pure SVD on single task and dual task walking. We hypothesized that (1) gait impairment in pure SVD would be most evident while dual task walking, (2) there is an association between processing speed, the main cognitive deficit in SVD, and gait, and (3) SVD‐related white matter alterations (as assessed by DTI) are associated with gait performance.

To our knowledge, this is the first study analyzing spatiotemporal gait data in patients with pure SVD. Here, we combine most recent methods of gait recording, diffusion tensor imaging and analysis.

## Methods

### Subjects

We included 39 CADASIL patients from an ongoing, prospective single‐center study in Munich, Germany. CADASIL was confirmed by either molecular genetic testing (sequencing of the *NOTCH3* gene) or by ultrastructural analysis of a skin biopsy (detection of pathognomonic granular osmiophilic material in vessel walls). Inclusion criteria were age ≤ 70 years, absence of focal neurological deficits (e.g., paresis after stroke), absence of signs for polyneuropathy, available data for gait, neuropsychological testing, and MRI. All examinations were performed within 2 consecutive days. One hundred and ninety‐two age‐ and sex‐matched healthy controls were recruited from local staff or by advertisement. In a standardized interview, none of the controls reported any auditory, vestibular, neurologic, cardiovascular, or orthopedic disorders. A short physical examination was performed to exclude impairments in motor and sensory functions, coordination, balance, orientation, and short‐term memory. All study participants had normal or corrected‐to‐normal vision. Leg length was measured in all subjects to be used as covariable in the statistical analysis. The study was performed in accordance with the Declaration of Helsinki and approved by the local ethics committee. Written informed consent was obtained from all subjects.

### Quantitative gait assessment

Spatiotemporal gait performance was assessed using the electronic, pressure‐sensitive GAITRite® carpet (CIR Systems, Havertown, USA) with a length of 670 cm and a sampling rate of 120 Hz. It was recorded under four different conditions similar to previously used experimental protocols.^13^, ^17^, ^18^, ^23^ Four trials were performed in each condition to increase the number of recorded gait cycles and thereby improving the reliability of gait parameters.^24^, ^25^ In the first condition, subjects were asked to walk over the carpet with preferred speed (condition 1, single task). In the remaining three conditions, subjects were asked to perform dual tasks. In the first dual task condition, subjects performed a calculatory cognitive task while walking (condition 2, calculatory dual task), that is, serial 7 task. Using this task, we tested the effect of a secondary, working memory task on gait.^26^ Next, subjects performed a semantic cognitive task while walking (condition 3, semantic dual task), that is, a verbal fluency task. This task was used to test the effect of a semantic memory task on gait.^27^ Finally, subjects performed a motoric, control task while walking (condition 4, motoric dual task), that is, carrying an empty tray. Subjects were asked to prioritize the secondary task during walking.

Each walk was started 150 cm in front of the carpet and continued for 150 cm beyond it in order to record steady‐state locomotion. Gait parameters were recorded as the mean of the four trials within each condition. We selected eight gait parameters that have been reported to correlate with cognitive deficits^8^ and/or neuroimaging aspects of SVD.^28^ These parameters can be assigned to three different domains.^19^ Parameters assigned to the pace domain were (1) velocity (cm/s), (2) cadence (steps/min), and (3) stride length (cm). Parameters assigned to the rhythm domain were (4) double support phase (% of gait cycle when both feet simultaneously have ground contact) and (5) swing phase (% of gait cycle when one foot is in the air). Parameters assigned to the variability domain were (6) stride time variability (%), (7) stride length variability (%), and (8) base of support variability (%). Variability was calculated as the coefficient of variation in percentage (CV = [standard deviation of parameter/mean of parameter] × 100). It represents the magnitude of stride‐to‐stride fluctuations within one gait parameter, with less variability suggesting higher gait automaticity and stability.^29^ All gait parameters were calculated with respect to the left leg side.

For analysis, we used single task walking performance and dual task costs, that is, the relative difference between dual task walking and single task walking (dual task costs = ([dual task walking – single task walking]/single task walking)  × 100). We assessed dual task costs in order to examine performance alterations under dual task walking in relation to single task walking.

Gait parameters in the single task and dual task costs were standardized. We transformed raw data into z‐scores by calculating means and standard deviations of 192 healthy controls (tested with the same gait protocol at our institution) separately for males and females in age ranges of 20–39, 40–59, and 60–79 years. We used *z*‐scores as an intuitive measure for effect size of differences between CADASIL patients and healthy controls. Negative *z*‐values represent worse performance compared to controls. A *z*‐value of 0 represents no difference between CADASIL patients and controls, that is, norm performance.

### Neuropsychological assessment

The Trail Making Test (TMT) is a paper–pencil test on executive functions, specifically mental flexibility and processing speed.^30^ In TMT matrix A, participants are asked to connect numbers presented at different locations on the sheet of paper from 1 to 25 in increasing order as quickly as possible. In TMT matrix B, numbers and letters have to be connected alternately in increasing order. TMT raw test scores were transformed into age‐ and education‐corrected z‐scores based on normative data from the literature.^31^ We prespecified processing speed for cognitive function analysis, because it is the most prominently and often only affected cognitive domain in SVD.^32^ More specifically, we used a previously established compound score of processing speed (mean z‐score of TMT A and B), which has been shown to highly correlate with white matter alterations in SVD.^33^, ^34^, ^35^

### Magnetic resonance imaging

MRI scans of all CADASIL patients were acquired on a single 3.0T Magnetom Verio scanner (Siemens Healthineers, Erlangen, Germany). The MRI protocol included 1 mm isotropic 3D‐T1, 1 mm isotropic 3D fluid‐attenuated inversion recovery (FLAIR), 2D‐T2 and diffusion MRI sequences (30 diffusion directions; b‐value 1000 sec/mm^2^, 2 mm isotropic). Complete details on sequence parameters have been described previously.^36^

The following SVD lesions were quantified according to the STRIVE consensus criteria^37^ to enable a better interpretation of sample characteristics: WMH volume, lacune volume, and brain volume. Processing pipelines have been described previously.^33^, ^38^ All volumes were normalized for head size by the intracranial volume.

### Diffusion tensor imaging

We used DTI to study the effect of white matter alterations on gait. DTI is a sensitive technique to characterize white matter microstructure by quantifying water diffusion in brain tissue.^39^ In SVD, the magnitude of diffusion in brain tissue is increased (increase in mean diffusivity, MD). To extract DTI measures, we performed the following processing steps:

After visual inspection to exclude major artifacts, diffusion data were preprocessed using MRtrix v3.0 package (http://www.mrtrix.org) and the Functional Magnetic Resonance Imaging of the Brain software library (FSL), v5.0.10.^40^ After noise and Gibbs ringing artifacts removal (“dwidenoise,”^41^ “mrdegibbs”;^42^ MRtrix), images were corrected for subject motion and eddy‐currents (“eddy_correct”; FSL). Diffusion tensors and scalar diffusion measures were estimated using “dtifit” (FSL).

We analyzed the effect of both global and regional white matter alterations on gait.

As a global measure for SVD‐related white matter alterations, we calculated the peak width of skeletonized mean diffusivity (PSMD).^35^ PSMD is a fully automated SVD burden marker and sensitively captures global alterations in white matter integrity. We prespecified PSMD as a marker for global white matter alterations, because it highly correlates with processing speed^33^, ^34^ and outperforms other MRI‐based markers (such as WMH volume, lacune volume, and brain volume) in explaining clinical deficits.^35^ PSMD was calculated with a publicly available script (http://www.psmd-marker.com).

To analyze regional white matter alterations, we calculated voxel‐wise MD values within major white matter tracts. We used the tract‐based spatial statistics (TBSS) pipeline^43^ within FSL with standard parameters and a fractional anisotropy standard template in Montreal Neurological Institute (MNI) space, provided by FSL. Rigorous checks were performed at each step of the pipeline. Finally, a custom mask was applied to exclude regions close to cerebrospinal fluid in order to avoid partial volume effects.

### Statistical analyses

Statistical analyses were performed in R (v3.4.1).^44^

Wilcoxon signed‐rank tests against zero were used to examine whether z‐scores (representing differences between CADASIL and healthy controls) were significantly different from zero, that is, from norm performance. We used nonparametric testing due to presence of nonnormally distributed values in patients.

The association between processing speed or global white matter alterations (assessed by PSMD) and gait performance was evaluated by multiple, linear regression models corrected for patients' leg lengths. Gait parameters were used as dependent variables. Gait parameters, processing speed scores, and PSMD values were power transformed in case of nonnormal distribution.

*P*‐values of multiple regressions and Wilcoxon signed‐rank tests against zero were Bonferroni‐corrected. Statistical significance level was set at α_corr._ < 0.05.

Regional associations between white matter alterations (voxel‐wise MD values as independent variables) and gait parameters (dependent variables) were performed using permutation test theory with a standard general linear model (“randomise”; FSL). All linear models were corrected for leg length. The number of permutations was set at 5000. Significant voxels within the skeletonized MD maps were identified using threshold‐free cluster enhancement with *P* < 0.05, corrected for multiple comparisons.

## Results

### Sample characteristics

**Figure 1:**
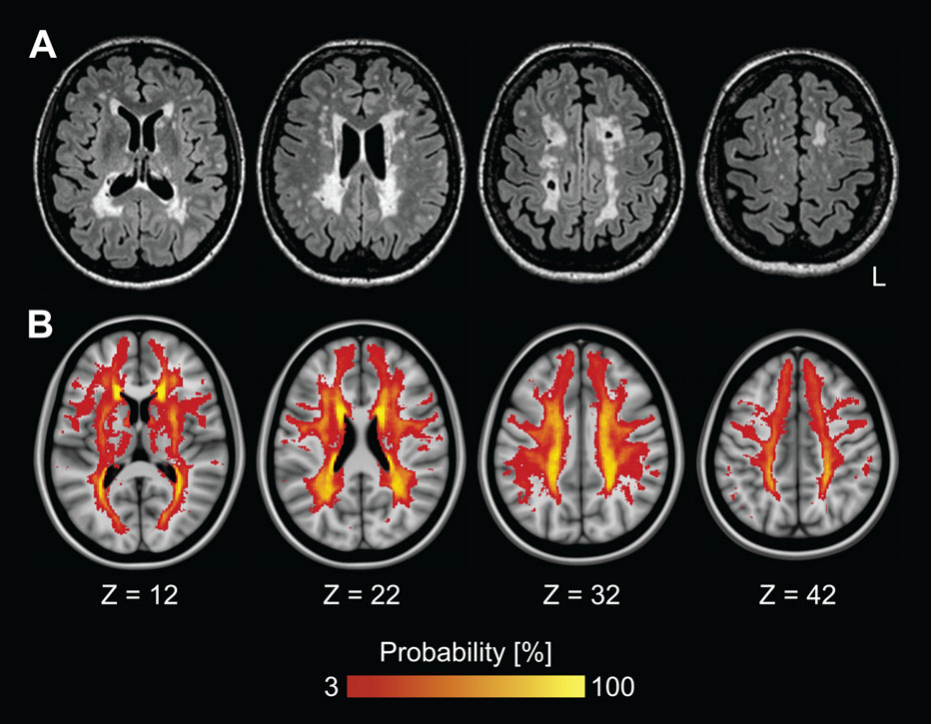
White matter hyperintensities in pure SVD. White matter hyperintensities on fluid‐attenuated inversion recovery (FLAIR) images. (A) Subject with median lesion load. (B) Lesion frequency map superimposed onto the Montreal Neurological Institute 152 standard brain template. L = left.

Sample characteristics are provided in Table 1. CADASIL patients showed a high WMH lesion load (Fig. 1). Raw values of single task gait performance and dual task costs for CADASIL patients as well as for healthy controls are depicted in Table 2.

**Table 1:** Sample characteristics.

	CADASIL *n* = 39
Demographic characteristics	
Age, years mean (SD) [min, max]	50.0 (8.1) [32.0, 62.0]
Education, years, mean (SD)	10.8 (1.6)
Female, No. [%]	27 [69]
Cognitive scores	
TMT‐A^1^, median (IQR) [min, max]	−0.22 (1.31) [−8.62, 1.29]
TMT‐B^1^, median (IQR) [min, max]	−0.43* (2.58) [−12.66, 1.72]
Processing speed^1^, median (IQR) [min, max]	−0.55** (2.06) [−10.64, 1.36]
Verbal fluency^1^, median (IQR) [min, max]	0.20 (1.39) [−1.83, 2.61]
MMSE, median (IQR) [min, max]	30 (1) [27, 30]
Imaging characteristics	
PSMD, 10^−4^ mm^2^/sec median (IQR) [min, max]	4.54 (2.32) [2.67, 9.21]
Normalized WMHV, %, median (IQR) [min, max]	4.40 (6.04) [0.09, 22.84]
Normalized LV, %, median (IQR) [min, max]	0.01 (0.06) [0.00, 0.25]
BPF, median (IQR) [min, max]	0.80 (0.06) [0.70, 0.87]

BPF, brain parenchymal fraction; IQR, interquartile range; LV, lacune volume; MMSE, Mini‐Mental State Examination; PSMD, peak width of skeletonized mean diffusivity; TMT, Trail Making Test; WMHV, white matter hyperintensity volume.

1 Age‐ and education‐adjusted *z*‐scores.

** *P*_corr._ < 0.01

* *P*_corr._ < 0.05; Wilcoxon signed‐rank tests against zero.

**Table 2:** Raw values of single task walking and dual task costs in CADASIL and healthy controls.

	CADASIL (*n* = 39)				Healthy controls (*n* = 192)			
	Single task	Dual task costs			Single task	Dual task costs		
		Calculatory	Semantic	Motoric		Calculatory	Semantic	Motoric
Pace								
Vel^1^ *cm/s*	115.0 (47.7) [56.8, 186.4]	−16.6 (24.8) [−59.3, 24.2]	−17.4 (18.4) [−61.3, 14.8]	−4.7 (19.5) [−47.6, 30.2]	113.6 (23.3) [74.5, 166.9]	−10.4 (17.2) [−39.7, 7.4]	−7.6 (16.8) [−33.0, 16.4]	3.9 (13.8) [−27.4, 27.5]
Cad^1^ *steps/min*	111.5 (15.6) [82.2, 138.1]	−8.7 (13.2) [−44.1, 13.9]	−9.3 (15.8) [−41.4, 4.7]	−0.2 (11.6) [−25.6, 15.7]	112.0 (13.0) [88.0, 132.4]	−5.3 (11.1) [−42.6, 9.9]	−5.0 (13.3) [−25.3, 8.1]	4.0 (7.7) [−12.4, 17.0]
SLen^1^ *cm*	127.8 (26.9) [73.0, 181.5]	−7.6 (10.5) [−38.2, 13.6]	−6.5 (12.5) [−33.7, 21.7]	−2.5 (10.9) [−30.7, 13.0]	125.0 (16.0) [98.2, 157.2]	−3.6 (10.7) [−20.4, 5.0]	0.5 (8.8) [−13.6, 9.4]	0.9 (9.2) [−18.0, 10.6]
Rhythm								
DSupp^1^ *%*	24.4 (5.7) [17.6, 34.4]	14.0 (18.4) [−12.1, 77.4]	11.6 (14.5) [−4.1, 47.2]	3.3 (12.8) [−10.1, 40.2]	21.3 (8.0) [13.0, 29.1]	5.0 (13.4) [−8.6, 912.8]	4.7 (13.9) [−9.7, 45.2]	−1.9 (12.9) [−12.3, 13.6]
Swing^1^ *%*	37.8 (2.4) [31.1, 40.4]	−3.7 (4.9) [−30.3, 4.7]	−2.5 (6.3) [−21.7, 3.5]	−1.3 (4.0) [−17.8, 8.1]	39.1 (5.1) [35.0, 43.8]	−1.4 (3.4) [−16.9, 5.2]	−0.2 (4.8) [−8.0, 6.2]	0.4 (4.0) [−7.4, 6.4]
Variability								
STime^1^ CV *%*	2.0 (1.4) [0.6, 7.8]	54.1 (147.4) [−78.3, 1005.0]	47.9 (165.6) [−83.5, 750.0]	−5.6 (62.3) [−67.4, 231.5]	1.7 (0.9) [0.8, 5.0]	94.3 (129.8) [−65.5, 1186]	6.4 (15.1) [−8.1, 32.2]	2.6 (61.1) [−75.8, 252.8]
SLen^1^ CV *%*	2.0 (1.6) [0.6, 10.7]	91.1 (93.3) [−51.1, 503.8]	81.0 (126.2) [−40.6, 425.2]	11.6 (97.6) [−63.0, 351.4]	2.3 (1.3) [0.8, 6.1]	38.1 (110.2) [−50.6, 330.3]	27.8 (71.5) [−56.5, 219.0]	−6.5 (65.3) [−69.1, 127.4]
BoS^1^ CV *%*	18.5 (11.0) [6.7, 57.0]	6.1 (63.2) [−60.2, 192.3]	−0.1 (74.1) [−66.8, 410.5]	−6.2 (71.5) [−56.0, 261.5]	20.1 (14.5) [6.8, 80.6]	−13.3 (98.3) [−94.2, 261.0]	−24.8 (67.2) [−148.8, 233.3]	−7.6 (64.7) [−77.5, 152.7]

BoS CV, base of support variability; Cad, cadence; DSupp, double support; SLen, stride length; STime CV, stride time variability; SLen CV, stride length variability; Swing, swing phase; Vel, velocity.

1 Median (interquartile range) [min, max].

### Moderate single task gait changes in the rhythm domain

**Figure 2:**
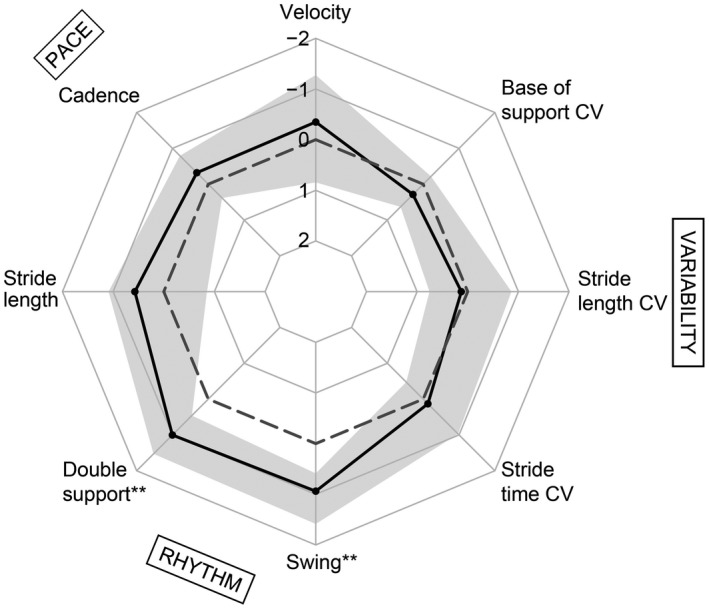
Single task walking. Median z‐values in CADASIL (solid black line) and interquartile ranges (gray) for single task walking parameters. Negative values represent worse performance compared to healthy controls (dashed norm line). **P_corr._ < 0.01, Wilcoxon signed‐rank tests against zero. CV, coefficient of variation.

Figure 2 shows the gait profile of CADASIL patients during single task walking. CADASIL patients performed worse than controls in the rhythm domain, that is, prolonged double support (*z*‐score median = −1.00; *P*_corr_ = 2.9 × 10^−7^) and shorter swing phase (*z*‐score median = −0.94; *P*_corr._ = 7.4 × 10^−9^). Of note, effect sizes were only modest with about one standard deviation. Other domains than the rhythm domain were not affected.

### Moderate increase in semantic dual task costs in the rhythm and pace domain

**Figure 3:**
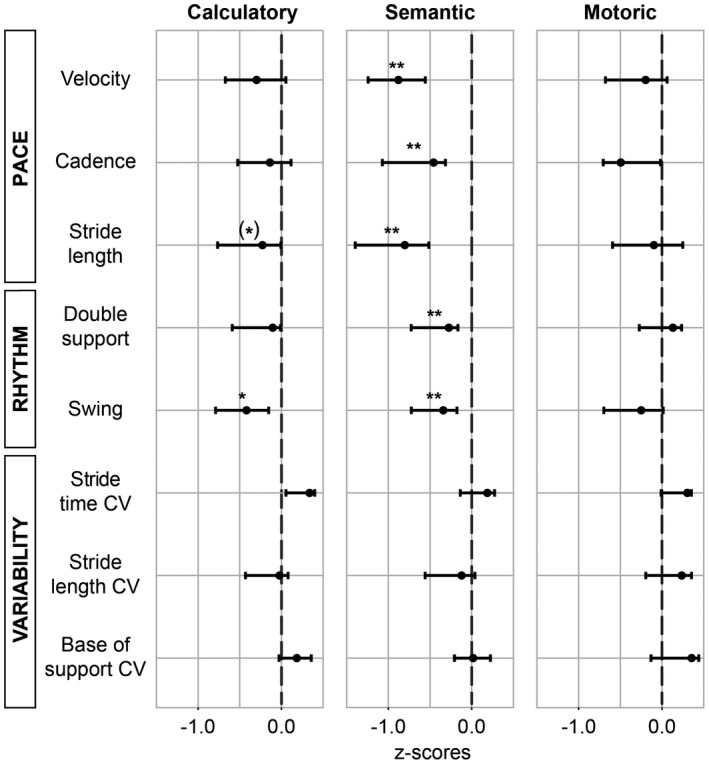
Dual task costs. Median *z*‐values (dots) of different dual task costs in CADASIL patients. Negative values represent higher costs compared to healthy controls (dashed norm line). **P_corr._ < 0.01, *P_corr._ < 0.05, (*) *P*_uncorr._ < 0.05; Wilcoxon signed‐rank tests against zero; bars depict the 95 % confidence interval. CV, coefficient of variation.

The effects of the calculatory, semantic, and motoric task on gait performance were assessed by dual task costs (Fig. 3). Gait performance was predominantly changed by the semantic task. More specifically, semantic dual task walking pronounced deficits in the rhythm domain, which had already been affected in single task walking (i.e., prolonged double support, *z*‐score median = −0.27; *P*_corr._ = 0.002, and swing phase, *z*‐score median = −0.34; *P*_corr._ = 0.005). In addition, the semantic task uncovered deficits in the pace domain (i.e., reduced gait velocity, z‐score median = −0.88; *P*_corr._ = 2.2 × 10^−5^, cadence, *z*‐score median = −0.46; *P*_corr._ = 0.002, and stride length, *z*‐score median = −0.80; *P*_corr._ = 3.3 × 10^−4^). The calculatory task affected swing phase only (*z*‐score median = −0.42; *P*_corr._ = 0.020). Again, effect sizes for dual task worsening were only moderate (less than one standard deviation of the performance in healthy subjects).

We did not find a dual‐tasking effect in the variability domain.

As expected, the motoric task, which has been used as a control task, did not worsen gait.

### Processing speed is not related to single task walking or dual task costs

Compared with healthy controls, CADASIL patients performed significantly worse in speed‐dependent cognitive tests (Table 1). Also, processing speed was significantly associated with global white matter alterations (PSMD) (β = −0.78, *R* ^2^_adj._ = 15.3 %; *P*_corr._ = 0.008). To investigate whether processing speed impacts on gait performance, we examined associations with single task walking or dual task costs. There was no significant association with any single task parameter or dual task costs (all *P*_uncorr._ > 0.051, all *P*_corr._ > 0.410).

### Global white matter alterations are associated with single task stride length

Finally, we examined the impact of SVD‐related white matter alterations on gait performance. First, we assessed the relationship between global white matter alteration (PSMD) and gait parameters. In the single task, higher PSMD was associated with shorter stride length (i.e., *β* = −0.21, *R* ^2^_adj._ = 18.0 %; *P*_corr._ = 0.030) (Table S1). The association between PSMD and single task velocity was marginally significant (*β* = −0.18, *R* ^2^_adj._ = 13.7 %; *P*_corr._ = 0.090). There was no association with any other single task parameter or dual task costs (all *P*_corr._ > 0.220).

**Figure 4:**
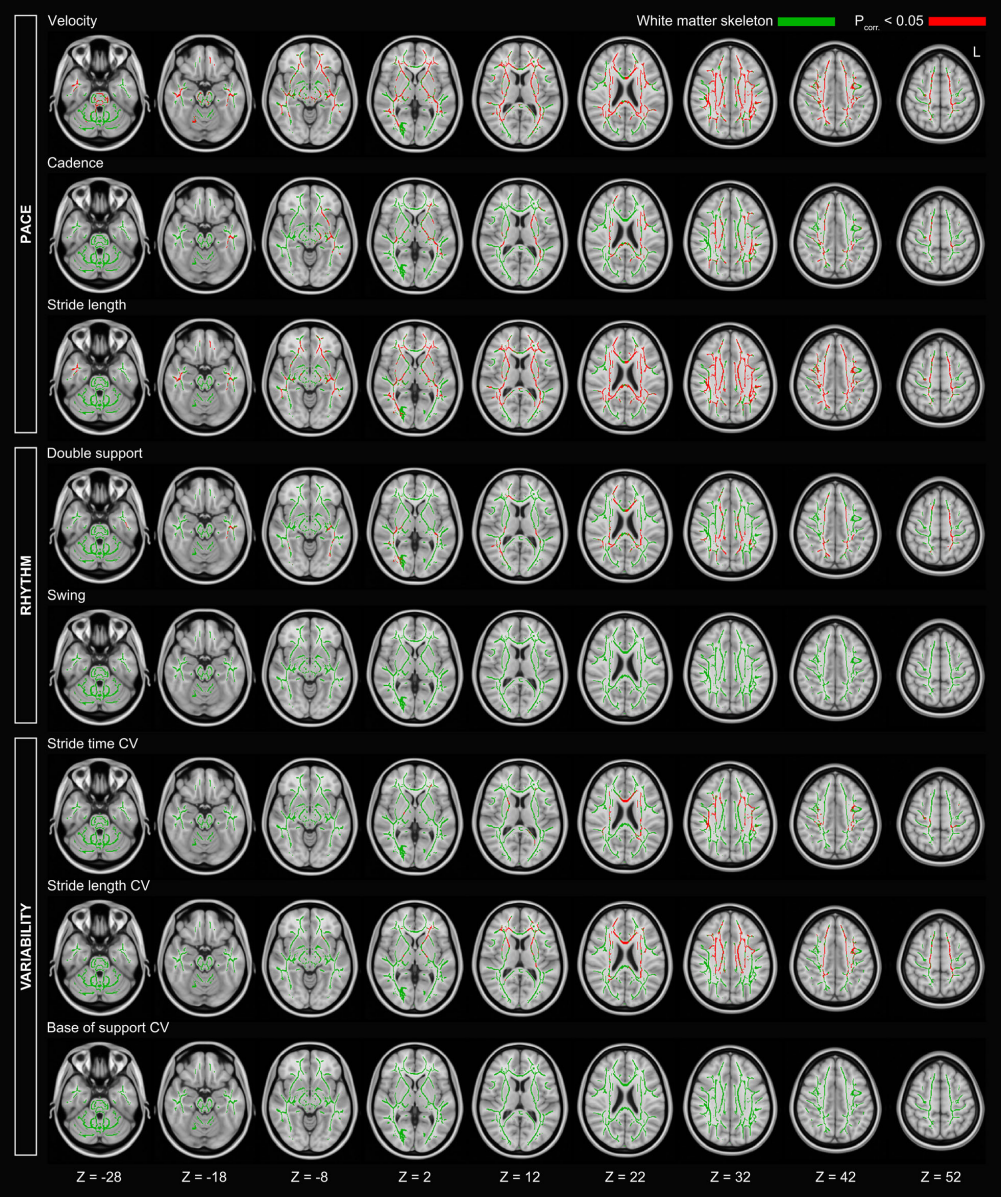
Voxel‐wise associations between mean diffusivity (MD) and single task walking. Axial slices of the white matter skeleton (green) superimposed onto the Montreal Neurological Institute 152 standard brain template. Depicted are significant associations (red) after correction for multiple comparisons. L = left.

Regional effects of white matter alterations (MD) on gait performance were assessed using voxel‐wise regression analyses. Higher MD values in the entire white matter skeleton were associated with shorter stride length and slower gait velocity in the single task (Fig. 4). Infratentorial white matter regions did not show significant voxels.

Significant associations were also found for single task cadence, double support, stride time variability, and stride length variability. For these gait parameters, instead of the entire skeleton being significant, we found smaller significant clusters (Fig. 4). Still, for these smaller clusters there was no clear preference for specific white matter tracts, as individual clusters were distributed over the entire white matter. No significant voxels were found for swing phase or base of support. Importantly, similar to the global analysis using PSMD, no significant regional associations were found for dual task costs.

## Discussion

We investigated the effect of pure, genetically defined SVD on gait while walking only (single task walking) and while performing a secondary cognitive or motoric task (dual task walking). We found that (1) despite severe brain lesions, single task gait performance in CADASIL patients was relatively preserved, with minor deficits only in the rhythm domain. (2) The semantic dual task aggravated gait rhythm deficits and uncovered pace deficits. (3) Cognitive impairment, that is, processing speed deficits, in pure SVD did not worsen gait and (4) global white matter alterations affected single task stride length only.

Gait performance of pure SVD patients with severe white matter alterations differed only slightly from that of healthy controls, more precisely around one standard deviation in the rhythm domain. Dual task walking, which has been used to uncover or pronounce gait deficits^3^ caused a moderate deterioration of the rhythm features and unmasked gait abnormalities in the pace domain that were not present during single task walking. Interestingly, we did not find an association between cognitive performance (i.e., processing speed) during neuropsychological testing and gait performance in our sample. Our results thus suggest that severe SVD alone and its effect on cognition might only play a minor role in causing gait impairment. In elderly, sporadic SVD patients, the combination with other age‐related pathologies might be decisive for gait decline. One might speculate that joint problems, sarcopenia, and reduced sensory input^3^ are therefore more promising targets for prevention and rehabilitation of gait deficits in the elderly. For instance, treatment for sarcopenia could include physical exercise, balance training, and protein supplementation to support muscle gain.^45^

### Affected gait domains in pure SVD

Although only moderate, differences in gait performance between pure SVD patients and healthy controls were detectable in the rhythm domain while single task walking. Changes in gait rhythm indicate difficulties in keeping balance and have been shown to correlate with SVD markers, like WMH.^4^

Semantic dual‐tasking pronounced gait deficits in the rhythm domain and additionally uncovered deficits in the pace domain, suggesting that brain networks that control rhythm and pace are interlinked with networks for the performance of the verbal fluency task. Control of gait rhythm and pace and the semantic task seem to compete for the same cognitive resources resulting in higher dual task costs. This is in line with previous studies showing that verbal fluency dual tasks resulted in reduction in gait velocity in community‐dwelling older adults,^5^ in individuals with MCI and Alzheimer's disease dementia.^18^

However, we did not find an effect of single or dual task walking on the variability domain in pure SVD patients indicating steady gait performance in all conditions. Gait variability has been described as a sensitive marker of dynamic gait stability and is an established parameter in fall risk assessment.^17^ It seems that our sample of pure SVD patients was able to engage enough cognitive resources to compensate increasing variability from stride‐to‐stride, even while cognitive dual‐tasking.

Ultimately, comparing affected gait domains or variables between studies with different cohorts is difficult, not only because of age differences and accompanying comorbidities in the study samples, but also because of the number and kind of examined gait variables (i.e., only velocity in most studies), and differences in secondary cognitive or motor tasks while walking. An agreement of standardized dual task methodologies is crucial in future research to further study gait impairment in SVD or neurodegenerative diseases.

### Differential effects of secondary cognitive tasks

In our sample of relatively young, pure SVD patients, the semantic (verbal fluency) dual task worsened gait more than the calculatory (serial 7) dual task. Some studies investigated the effect of type and complexity of the secondary tasks on dual task walking.^17^, ^18^, ^23^, ^46^ Contrary to our results, it has been shown that the serial 7 task generates greater cognitive load than verbal fluency tasks in frail, older adults and subjects with MCI and Alzheimer's disease, resulting in worse gait performance in the calculatory than the semantic task. A possible explanation for this difference with previous studies might be the typical cognitive profile in SVD, with deficits predominantly in processing speed. Verbal fluency is a semantic memory task imposing substantial demands upon processing speed during retrieval from semantic long‐term memory.

### Effect of processing speed on gait

Other than expected, we did not find an association between processing speed deficits and single task gait or dual task costs in pure SVD. Cognitive deficits, beside white matter alterations, are thought to be an important factor for gait disturbances, for example, as shown in frail older adults, individuals with MCI, and demented patients while single task and dual task walking.^13^, ^14^, ^17^, ^18^, ^47^, ^48^, ^49^ Yet, the effect of cognitive impairment on single and dual task gait has not been examined specifically in SVD. Our results in pure SVD patients suggest that cognitive deficits related to SVD do not worsen gait. Of note, none of our subjects was demented and thus we cannot exclude a detrimental effect of cognition on gait in late disease stages. Generalizability of existing study results about the relation between cognition and gait is limited, as tests used to measure processing speed or executive function vary between studies. Also, examined samples are considerably older than ours and the presence of age‐related pathologies was not always systematically assessed or excluded. The possibility remains that in previous studies associations between cognitive deficits and gait were at least in part driven by age‐related comorbidities.

### Effect of white matter alterations on gait

Correlation analyses revealed significant associations between SVD‐related white matter alterations measured by DTI and reduced stride length and marginally velocity in pure SVD patients. The same parameters were affected in sporadic SVD patients with strategic brain lesions related to gait deficits.^6^, ^9^ Using the voxel‐wise analysis, we found no indication for regional effects or spatial heterogeneity. Instead, we found a rather global effect of supratentorial white matter alterations on pace parameters, that is, on stride length and velocity, but not on cadence. In line with a study by de Laat and colleagues,^6^ only few voxels with higher MD were related to a lower cadence, suggesting that the control of cadence is less affected by white matter alterations than other pace parameters. Thus, white matter alterations might predominantly influence spatial characteristics of stepping like stride length. Temporal pace maker regions in the locomotor network, such as the cerebellar locomotor regions, do not appear to be affected in pure SVD.

### Limitations

Some limitations need to be considered. First, we did not obtain MRI in our control group, therefore subclinical SVD cannot be excluded in our clinically healthy sample. Second, while the examination of eight different spatiotemporal gait variables allowed precise description of gait performance, correcting for multiple comparisons is accompanied by a loss of statistical power. Third, our results are based on cross‐sectional data, which do not allow to draw conclusions on causality. While our sample of pure SVD patients was relatively small, it enabled to detect subtle differences between groups and provided the unique opportunity to study the effects of pure SVD.

## Conclusion

Despite severe brain lesions in genetically defined, pure SVD patients, gait performance was relatively preserved. Differences between pure SVD patients and healthy controls in single task walking and dual task costs were only moderate. Neither processing speed performance nor white matter alterations were associated with dual task costs. We speculate that other age‐related morbidities affecting the brain or other relevant organ systems, such as neurodegeneration, pharmacotherapy, sarcopenia, musculoskeletal disease, or polyneuropathy contribute toward gait impairment in elderly people. These factors should be considered in future research, as well as in new strategies for intervention and rehabilitation.

## Author Contribution

Sofia Finsterwalder contributed to the design and execution of data analysis, wrote the first draft, and prepared figures. Max Wuehr contributed to the conception of the research project, the preparation of statistical analysis, and revised the manuscript. Benno Gesierich contributed to data analysis, preparation of figures, and revised the manuscript. Anna Dietze contributed to the execution of data analysis. Marek J. Konieczny contributed to imaging data analysis and revised the manuscript. Reinhold Schmidt contributed to the interpretation of the data and revised the manuscript. Roman Schniepp contributed to the conception, organization, and execution of the research project, and revised the manuscript. Marco Duering contributed to the conception, organization, and execution of the research project, conceptualized statistical analysis, and revised the manuscript.

## Conflict of Interest

We report no relevant conflict of interest. MD reports personal fees from Bayer Vital GmbH and from Pfizer Pharma GmbH outside the submitted work.

## Supporting information


**Table S1**. Linear regressions with global white matter alterations (PSMD) in the single task.
