# Supplementary material for: Minor gait impairment despite white matter damage in pure small vessel disease
Source: Ann Clin Transl Neurol. 2019 Sep 16;6(10):2026–36. doi: 10.1002/acn3.50891 (PMC6801180; doi:10.1002/acn3.50891)
Supplement: Supplementary file 1 — Table S1. Linear regressions with global white matter alterations (PSMD) in the single task. [file ACN3-6-2026-s001.pdf]

Supplementary Table 1. Linear regressions with global white matter alterations (PSMD) in the single task

**Regressor Single task**	**β**	$P_{uncorr.}$	$P_{corr.}$	R^2 adj. [%]
**Pace**				
Vel	-0.176	0.01	0.09	13.7
Cad	-0.148	0.08	0.65	5.5
SLen	-0.207	0.00	0.03*	18.0
**Rhythm**				
DSupp	-0.185	0.08	0.62	5.7
Swing	-0.190	0.33	1.00	0.0
**Variability**				
STime CV	-0.273	0.03	0.24	9.7
SLen CV	-0.192	0.04	0.22	8.5
BoS CV	-0.027	0.84	1.00	0.0

*$P_{corr.} < 0.05$; Linear regressions corrected for leg length.

BoS CV, base of support variability; Cad, cadence; DSupp, double support; SLen, stride length; SLen CV, stride length variability; STime CV, stride time variability; Swing, swing phase; Vel, velocity.
